# Approximation by $(p,q)$-Lupaş–Schurer–Kantorovich operators

**DOI:** 10.1186/s13660-018-1858-9

**Published:** 2018-09-26

**Authors:** Kadir Kanat, Melek Sofyalıoğlu

**Affiliations:** Ankara Hacı Bayram Veli University Polatlı Faculty of Science and Arts, Ankara, Turkey

**Keywords:** Lupaş operators, $(p,q)$-integers, Rate of convergence, Local approximation, Korovkin’s approximation theorem

## Abstract

In the current paper, we examine the $(p,q)$-analogue of Kantorovich type Lupaş–Schurer operators with the help of $(p,q)$-Jackson integral. Then, we estimate the rate of convergence for the constructed operators by using the modulus of continuity in terms of a Lipschitz class function and by means of Peetre’s K-functionals based on Korovkin theorem. Moreover, we illustrate the approximation of the $(p,q)$-Lupaş–Schurer–Kantorovich operators to appointed functions by the help of Matlab algorithm and then show the comparison of the convergence of these operators with Lupaş–Schurer operators based on $(p,q)$-integers.

## Introduction

In 1962, Bernstein–Schurer operators were identified in the paper of Schurer [25]. In 1987, Lupaş [16] initiated the *q*-generalization of Bernstein operators in rational form. Some other *q*-Bernstein polynomial was defined by Phillips [22] in 1997. The development *q*-calculus applications established a precedent in the field of approximation theory. We may refer to some of them as Durrmeyer variant of *q*-Bernstein–Schurer operators [2], *q*-Bernstein–Schurer–Kantorovich type operators [3], *q*-Durrmeyer operators [8], *q*-Bernstein–Schurer–Durrmeyer type operators [12], *q*-Bernstein–Schurer operators [19], King’s type modified *q*-Bernstein–Kantorovich operators [20], *q*-Bernstein–Schurer–Kantorovich operators [23]. Lately, Mursaleen et al. [17] pioneered the research of $(p,q)$-analogue of Bernstein operators which is a generalization of *q*-Bernstein operators (Philips). The application of $(p,q)$-calculus has led to the discovery of various modifications of Bernstein polynomials involving $(p,q)$-integers. For instance, Mursaleen et al. [18] constructed $(p,q)$-analogue of Bernstein-Kantorovich operators in 2016, and Khalid et al. [15] generalised *q*-Bernstein–Lupaş operators. In the $(p,q)$-calculus, parameter *p* provides suppleness to the approximation. Some recent articles are [1, 4–6, 9, 10, 13], and [21]. Motivated by the work of Khalid et al. [15], now we define a Kantorovich type Lupaş-Schurer operators based on the $(p,q)$-calculus.

First of all, we introduce some important notations and definitions for the $(p,q)$-calculus, which is a generalization of *q*-oscillator algebras. For $0< q< p\leq1$ and $m\geq0$, the $(p,q)$-number of *m* is denoted by $[m]_{p,q}$ and is defined by

$$ [m]_{p,q}:=p^{m-1}+p^{m-2}q+\cdots+pq^{m-2}+q^{m-1}= \textstyle\begin{cases} \frac{p^{m}-q^{m}}{p-q} & \text{if } p\neq q\neq1, \\ \frac{1-q^{m}}{1-q} & \text{if } p= 1, \\ m & \text{if } p=q=1. \end{cases} $$

The formula for the $(p,q)$-binomial expansion is defined by

1 $${\left(cx+dy\right)}_{p,q}^{m}:=\sum_{l=0}^{m}{\left[\begin{array}{c} m\\ l \end{array}\right]}_{p,q}p^{\frac{\left(m-l)(m-l-1\right)}{2}}q^{\frac{l(l-1)}{2}}c^{m-l}d^{l}x^{m-l}y^{l},$$

where

$${\left[\begin{array}{c} m\\ l \end{array}\right]}_{p,q}=\frac{{\left[m\right]}_{p,q}!}{{\left[l\right]}_{p,q}!{\left[m-l\right]}_{p,q}!}$$

are the $(p,q)$-binomial coefficients. From Eq. (1) we get

$$ (x+y)_{p,q}^{m} =(x+y) (px+qy) \bigl(p^{2}x+q^{2}y \bigr)\cdots \bigl(p^{m-1}x+q^{m-1}y \bigr) $$

and

$$ (1-x)_{p,q}^{m} =(1-x) (p-qx) \bigl(p^{2}-q^{2}x \bigr)\cdots \bigl(p^{m-1}-q^{m-1}x \bigr). $$

The $(p,q)$-Jackson integrals are defined by

$$ \int_{0}^{a}f(x)\,d_{p,q}x=(q-p)a \sum _{k=0}^{ \infty} \frac{p^{k}}{q^{k+1}}f \biggl( \frac{p^{k}}{q^{k+1}}a \biggr), \quad \biggl\vert \frac{p}{q} \biggr\vert < 1 $$

and

$$ \int_{0}^{a}f(x) \,d_{p,q}x=(p-q)a \sum _{k=0}^{ \infty} \frac{q^{k}}{p^{k+1}}F \biggl( \frac{q^{k}}{p^{k+1}}a \biggr),\quad \biggl\vert \frac{q}{p} \biggr\vert < 1. $$

For detailed information about the theory of $(p,q)$-integers, we refer to [11] and [24].

## Construction of the operator

### Definition 1

For any $0< q< p\leq1$, we construct a $(p,q)$-analogue of Kantorovich type Lupaş–Schurer operator by

2 $$ K_{m,s}^{(p,q)}(f;x)=[m]_{p,q}\sum _{l=0}^{m+s}\frac{B_{m,l,s}^{p,q}(x)}{p^{m-l}q^{l}} \int_{\frac{[l]_{p,q}}{p^{l-m-1}[m]_{p,q}}}^{\frac {[l+1]_{p,q}}{p^{l-m}[m]_{p,q}}} f(t)\,d_{p,q}t,\quad x \in [0,1], $$

where $m\in\mathbb{N}$, $f\in C[0,s+1]$, $s>0$ is a fixed natural number and

3 $$B_{m,l,s}^{p,q}(x)=\frac{{\left[\begin{array}{c} m+s\\ l \end{array}\right]}_{p,q}p^{\frac{\left(m+s-l)(m+s-l-1\right)}{2}}q^{\frac{l(l-1)}{2}}x^{l}{\left(1-x\right)}^{m+s-l}}{\prod_{j=1}^{m+s}\{p^{j-1}(1-x)+q^{j-1}x\}}.$$

After some calculations we obtain

4 $$ K_{m,s}^{(p,q)}(f;x)=\sum _{l=0}^{m+s}B_{m,l,s}^{p,q}(x) \int_{0}^{1}f \biggl( \frac{p[l]_{p,q}+q^{l}t}{p^{l-m}[m]_{p,q}} \biggr) \,d_{p,q}t. $$

In the following lemma, we present some equalities for the $(p,q)$-analogue of Lupaş–Schurer–Kantorovich operators.

### Lemma 1

*Let* $K_{m,s}^{(p,q)}(\cdot;\cdot)$ *be given by Eq*. (4). *Then we have*

5 $$\begin{aligned}& K_{m,s}^{(p,q)}(1;x) = 1, \end{aligned}$$

6 $$\begin{aligned}& K_{m,s}^{(p,q)}(t;x) = \biggl( \frac{[m+s]_{p,q}}{[m]_{p,q}p^{s-1}} - \frac{p^{m}}{[2]_{p,q}[m]_{p,q}}+ \frac{q^{m+s}}{[2]_{p,q}[m]_{p,q}p^{s}} \biggr)x+\frac{p^{m}}{[2]_{p,q}[m]_{p,q}}, \end{aligned}$$

7 $$\begin{aligned}& \begin{aligned}[b] K_{m,s}^{(p,q)} \bigl(t^{2};x \bigr) &= \frac {[m+s]_{p,q}[m+s-1]_{p,q}q^{2}p^{2-2s}}{[m]_{p,q}^{2}(p(1-x)+qx)}x^{2}+\frac {[m+s]_{p,q}p^{m-s+1}}{[m]_{p,q}^{2}}x \\ &\quad {}+\frac {2[m+s]_{p,q}qp^{4m+2s-3}(p^{m+s}(1-x)+q^{m+s}x)}{[2]_{p,q}[m]_{p,q}^{2}(p(1-x)+qx)}x \\ &\quad {}+\frac {p^{-2s}(p^{m+s}(1-x)+q^{m+s}x)(p^{m+s+1}(1-x)+q^{m+s+1}x)}{[3]_{p,q}[m]_{p,q}^{2}(p(1-x)+qx)}, \end{aligned} \end{aligned}$$

8 $$\begin{aligned}& K_{m,s}^{(p,q)}(t-x;x) = \biggl( \frac{[m+s]_{p,q}}{[m]_{p,q}p^{s-1}} - \frac{p^{m}}{[2]_{p,q}[m]_{p,q}}+ \frac{q^{m+s}}{[2]_{p,q}[m]_{p,q}p^{s}}-1 \biggr)x+\frac{p^{m}}{[2]_{p,q}[m]_{p,q}}, \end{aligned}$$

9 $$\begin{aligned}& \begin{aligned}[b] K_{m,s}^{(p,q)} \bigl((t-x)^{2};x \bigr) &= \biggl( \frac {[m+s]_{p,q}[m+s-1]_{p,q}q^{2}p^{2-2s}}{[m]_{p,q}^{2}(p(1-x)+qx)} \\ &\quad {}+\frac {-2[2]_{p,q}[m+s]_{p,q}p^{1-s}+2p^{m}-2q^{m+s}p^{-s}}{[2]_{p,q}[m]_{p,q}}+1 \biggr) x^{2} \\ &\quad {}+ \biggl(\frac{[m+s]_{p,q}p^{m-s+1}}{[m]_{p,q}^{2}} +\frac {2[m+s]_{p,q}qp^{4m+2s-3}(p^{m+s}(1-x)+q^{m+s}x)}{[2]_{p,q}[m]_{p,q}^{2}(p(1-x)+qx)}\hspace{-20pt} \\ &\quad {}-\frac{2p^{m}}{[2]_{p,q}[m]_{p,q}} \biggr)x \\ &\quad {}+ \frac {p^{-2s}(p^{m+s}(1-x)+q^{m+s}x)(p^{m+s+1}(1-x)+q^{m+s+1}x)}{[3]_{p,q}[m]_{p,q}^{2}(p(1-x)+qx)}. \end{aligned} \end{aligned}$$

### Proof

*(i)* From the definition of the operators in (4), we can easily prove the first claim as follows:

10 $$\begin{array}{rcl} K_{m,s}^{\left(p,q\right)}(1;x) & = & \sum_{l=0}^{m+s}B_{m,l,s}^{p,q}(x)\int_{0}^{1}d_{p,q}t\\ & = & \sum_{l=0}^{m+s}\frac{{\left[\begin{array}{c} m+s\\ l \end{array}\right]}_{p,q}p^{\frac{\left(m+s-l)(m+s-l-1\right)}{2}}q^{\frac{l(l-1)}{2}}x^{l}{\left(1-x\right)}^{m+s-l}}{\prod_{j=1}^{m+s}\{p^{j-1}(1-x)+q^{j-1}x\}}\\ & = & 1. \end{array}$$

*(ii)* We can calculate the second identity for $K_{m,s}^{(p,q)}(t;x)$ as follows:

$$\begin{aligned} K_{m,s}^{(p,q)}(t;x) =&\sum_{l=0}^{m+s}B_{m,l,s}^{p,q}(x) \int_{0}^{1} \frac{p[l]_{p,q}+q^{l}t}{p^{l-m}[m]_{p,q}} \,d_{p,q}t \\ =&\sum_{l=0}^{m+s}B_{m,l,s}^{p,q}(x) \frac{p[l]_{p,q}}{p^{l-m}[m]_{p,q}} \int_{0}^{1} d_{p,q}t+\sum _{l=0}^{m+s}B_{m,l,s}^{p,q}(x) \frac{q^{l}}{p^{l-m}[m]_{p,q}} \int_{0}^{1} t \,d_{p,q}t . \end{aligned}$$

After that, by some simple computations, we have

$$\begin{array}{rcl} K_{m,s}^{\left(p,q\right)}(t;x) & = & \sum_{l=0}^{m+s}B_{m,l,s}^{p,q}(x)\frac{p{\left[l\right]}_{p,q}}{p^{l-m}{\left[m\right]}_{p,q}}+\sum_{l=0}^{m+s}B_{m,l,s}^{p,q}(x)\frac{q^{l}}{p^{l-m}{\left[m\right]}_{p,q}{\left[2\right]}_{p,q}}\\ & = & \sum_{l=1}^{m+s}\frac{p^{m-l+1}{\left[m+s\right]}_{p,q}}{{\left[m\right]}_{p,q}}.\frac{{\left[\begin{array}{c} m+s-1\\ l-1 \end{array}\right]}_{p,q}p^{\frac{\left(m+s-l)(m+s-l-1\right)}{2}}q^{\frac{l(l-1)}{2}}x^{l}{\left(1-x\right)}^{m+s-l}}{\prod_{j=1}^{m+s}\{p^{j-1}(1-x)+q^{j-1}x\}}\\ & & +\frac{1}{{\left[m\right]}_{p,q}{\left[2\right]}_{p,q}p^{s}}\sum_{l=0}^{m+s}\frac{{\left[\begin{array}{c} m+s\\ l \end{array}\right]}_{p,q}p^{\frac{\left(m+s-l)(m+s-l-1\right)}{2}}q^{\frac{l(l-1)}{2}}{\left(\frac{qx}{p(1-x)}\right)}^{l}}{\prod_{j=0}^{m+s-1}\{p^{j-1}+q^{j-1}(\frac{qx}{p(1-x)})\}}\\ & = & \frac{{\left[m+s\right]}_{p,q}}{{\left[m\right]}_{p,q}p^{s}}\sum_{l=0}^{m+s-1}\frac{p^{m+s-l}{\left[\begin{array}{c} m+s-1\\ l \end{array}\right]}_{p,q}p^{\frac{\left(m+s-l-1)(m+s-l-2\right)}{2}}q^{\frac{l(l+1)}{2}}x^{l+1}{\left(1-x\right)}^{m+s-l-1}}{\prod_{j=1}^{m+s-1}\{p^{j}(1-x)+q^{j}x\}}\\ & & +\frac{p(1-x)\{p^{m+s-1}+q^{m+s-1}(\frac{qx}{p(1-x)})\}}{{\left[m\right]}_{p,q}{\left[2\right]}_{p,q}p^{s}}\\ & & \times \sum_{l=0}^{m+s}\frac{{\left[\begin{array}{c} m+s\\ l \end{array}\right]}_{p,q}p^{\frac{\left(m+s-l)(m+s-l-1\right)}{2}}q^{\frac{l(l-1)}{2}}{\left(\frac{qx}{p(1-x)}\right)}^{l}}{\prod_{j=1}^{m+s}\{p^{j-1}+q^{j-1}(\frac{qx}{p(1-x)})\}}\\ & = & \frac{{\left[m+s\right]}_{p,q}}{{\left[m\right]}_{p,q}p^{s-1}}x+\frac{p(1-x)\{p^{m+s-1}+q^{m+s-1}(\frac{qx}{p(1-x)})\}}{{\left[m\right]}_{p,q}{\left[2\right]}_{p,q}p^{s}}. \end{array}$$

Then, $K_{m,s}^{(p,q)}(t;x)$ is obtained as

$$ K_{m,s}^{(p,q)}(t;x)= \biggl( \frac{[m+s]_{p,q}}{[m]_{p,q}p^{s-1}} - \frac{p^{m}}{[2]_{p,q}[m]_{p,q}}+ \frac{q^{m+s}}{[2]_{p,q}[m]_{p,q}p^{s}} \biggr)x+\frac{p^{m}}{[2]_{p,q}[m]_{p,q}}. $$

Thus, (6) is obtained.

*(iii)* For the third identity involving $K_{m,s}^{(p,q)}(t^{2};x)$, we write

11 $$\begin{aligned} K_{m,s}^{(p,q)} \bigl(t^{2};x \bigr) =&\sum _{l=0}^{m+s}B_{m,l,s}^{p,q}(x) \frac{p^{2}[l]^{2}_{p,q}}{p^{2l-2m}[m]^{2}_{p,q}} \int_{0}^{1} d_{p,q}t+2\sum _{l=0}^{m+s}B_{m,l,s}^{p,q}(x) \frac{p[l]_{p,q}q^{l}}{p^{2l-2m}[m]^{2}_{p,q}} \int_{0}^{1} t \,d_{p,q}t \\ &{}+\sum_{l=0}^{m+s}B_{m,l,s}^{p,q}(x) \frac{q^{2l}}{p^{2l-2m}[m]^{2}_{p,q}} \int_{0}^{1} t^{2} \,d_{p,q}t \\ =& \underbrace{\sum_{l=0}^{m+s}B_{m,l,s}^{p,q}(x) \frac{p^{2}[l]^{2}_{p,q}}{p^{2l-2m}[m]^{2}_{p,q}}}_{\text{B1}} + \underbrace {\frac{2}{[2]_{p,q}}\sum _{l=0}^{m+s}B_{m,l,s}^{p,q}(x) \frac{p[l]_{p,q}q^{l}}{p^{2l-2m}[m]^{2}_{p,q}}}_{\text{B2}} \\ &{}+ \underbrace{\frac{1}{[3]_{p,q}}\sum_{l=0}^{m+s}B_{m,l,s}^{p,q}(x) \frac{q^{2l}}{p^{2l-2m}[m]^{2}_{p,q}}}_{\text{B3}}. \end{aligned}$$

Firstly, we calculate *B*1 as

$$\begin{array}{rcl} B1 & = & \sum_{l=0}^{m+s}B_{m,l,s}^{p,q}(x)\frac{p^{2}{\left[l\right]}_{p,q}^{2}}{p^{2l-2m}{\left[m\right]}_{p,q}^{2}}\\ & = & \sum_{l=0}^{m+s-1}\frac{p^{2m-2l}{\left[l+1\right]}_{p,q}{\left[m+s\right]}_{p,q}}{{\left[m\right]}_{p,q}^{2}}.\frac{{\left[\begin{array}{c} m+s-1\\ l \end{array}\right]}_{p,q}p^{\frac{\left(m+s-l-1)(m+s-l-2\right)}{2}}q^{\frac{l(l+1)}{2}}x^{l+1}{\left(1-x\right)}^{m+s-l-1}}{\prod_{j=1}^{m+s}\{p^{j-1}(1-x)+q^{j-1}x\}}. \end{array}$$

Now by using the equality

12 $$ [l+1]_{p,q}=p^{l}+q[l]_{p,q}, $$

we acquire

13 $$\begin{array}{rcl} B1 & = & \frac{{\left[m+s\right]}_{p,q}}{{\left[m\right]}_{p,q}^{2}}\sum_{l=0}^{m+s-1}p^{2m-l}\frac{{\left[\begin{array}{c} m+s-1\\ l \end{array}\right]}_{p,q}p^{\frac{\left(m+s-l-1)(m+s-l-2\right)}{2}}q^{\frac{l(l+1)}{2}}x^{l+1}{\left(1-x\right)}^{m+s-l-1}}{\prod_{j=1}^{m+s}\{p^{j-1}(1-x)+q^{j-1}x\}}\\ & & +\frac{{\left[m+s\right]}_{p,q}}{{\left[m\right]}_{p,q}^{2}}\sum_{l=0}^{m+s-1}p^{2m-2l}q{\left[l\right]}_{p,q}\frac{{\left[\begin{array}{c} m+s-1\\ l \end{array}\right]}_{p,q}p^{\frac{\left(m+s-l-1)(m+s-l-2\right)}{2}}q^{\frac{l(l+1)}{2}}x^{l+1}{\left(1-x\right)}^{m+s-l-1}}{\prod_{j=1}^{m+s}\{p^{j-1}(1-x)+q^{j-1}x\}}\\ & = & \frac{{\left[m+s\right]}_{p,q}p^{2m}x}{{\left[m\right]}_{p,q}^{2}p^{m+s-1}}\sum_{l=0}^{m+s-1}\frac{{\left[\begin{array}{c} m+s-1\\ l \end{array}\right]}_{p,q}p^{\frac{\left(m+s-l-1)(m+s-l-2\right)}{2}}q^{\frac{l(l-1)}{2}}{\left(\frac{qx}{p(1-x)}\right)}^{l}{\left(1-x\right)}^{m+s-1}}{\frac{1}{p^{m+s-1}}\prod_{j=1}^{m+s-1}\{p^{j}(1-x)+q^{j}x\}}\\ & & +\frac{{\left[m+s\right]}_{p,q}{\left[m+s-1\right]}_{p,q}q^{2}x^{2}}{{\left[m\right]}_{p,q}^{2}p^{2s-2}(p(1-x)+qx)}\sum_{l=0}^{m+s-2}\frac{{\left[\begin{array}{c} m+s-2\\ l \end{array}\right]}_{p,q}p^{\frac{\left(m+s-l-2)(m+s-l-3\right)}{2}}q^{\frac{l(l-1)}{2}}{\left(\frac{q^{2}x}{p^{2}(1-x)}\right)}^{l}}{\prod_{j=1}^{m+s-2}\{p^{j-1}+q^{j-1}(\frac{q^{2}x}{p^{2}(1-x)})\}}\\ & = & \frac{{\left[m+s\right]}_{p,q}p^{m-s+1}}{{\left[m\right]}_{p,q}^{2}}x+\frac{{\left[m+s\right]}_{p,q}{\left[m+s-1\right]}_{p,q}p^{2-2s}q^{2}}{{\left[m\right]}_{p,q}^{2}(p(1-x)+qx)}x^{2}. \end{array}$$

Secondly, we work out *B*2 as follows:

14 $$\begin{array}{rcl} B2 & = & \frac{2}{{\left[2\right]}_{p,q}}\sum_{l=0}^{m+s}B_{m,l,s}^{p,q}(x)\frac{p{\left[l\right]}_{p,q}q^{l}}{p^{2l-2m}{\left[m\right]}_{p,q}^{2}}\\ & = & \frac{2{\left[m+s\right]}_{p,q}}{{\left[2\right]}_{p,q}{\left[m\right]}_{p,q}^{2}}\sum_{l=1}^{m+s}\frac{q^{l}}{p^{2l-2m-1}}.\frac{{\left[\begin{array}{c} m+s-1\\ l-1 \end{array}\right]}_{p,q}p^{\frac{\left(m+s-l)(m+s-l-1\right)}{2}}q^{\frac{l(l-1)}{2}}x^{l}{\left(1-x\right)}^{m+s-l}}{\prod_{j=1}^{m+s}\{p^{j-1}(1-x)+q^{j-1}x\}}\\ & = & \frac{2{\left[m+s\right]}_{p,q}x}{{\left[2\right]}_{p,q}{\left[m\right]}_{p,q}^{2}}\sum_{l=0}^{m+s-1}\frac{q}{p^{-2m+1}}.\frac{{\left[\begin{array}{c} m+s-1\\ l \end{array}\right]}_{p,q}p^{\frac{\left(m+s-l-1)(m+s-l-2\right)}{2}}q^{\frac{l(l-1)}{2}}{\left(\frac{q^{2}x}{p^{2}(1-x)}\right)}^{l}{\left(1-x\right)}^{m+s-1}}{\prod_{j=2}^{m+s}\{p^{j-1}(1-x)+q^{j-1}x\}}\\ & = & \frac{2{\left[m+s\right]}_{p,q}qp^{2m-1}x}{{\left[2\right]}_{p,q}{\left[m\right]}_{p,q}^{2}}\sum_{l=0}^{m+s-1}\frac{{\left[\begin{array}{c} m+s-1\\ l \end{array}\right]}_{p,q}p^{\frac{\left(m+s-l-1)(m+s-l-2\right)}{2}}q^{\frac{l(l-1)}{2}}{\left(\frac{q^{2}x}{p^{2}(1-x)}\right)}^{l}{\left(1-x\right)}^{m+s-1}}{\prod_{j=0}^{m+s-2}\{p^{j+1}(1-x)+q^{j+1}x\}}\\ & = & \frac{2{\left[m+s\right]}_{p,q}qp^{4m+2s-3}x}{{\left[2\right]}_{p,q}{\left[m\right]}_{p,q}^{2}}\sum_{l=0}^{m+s-1}\frac{{\left[\begin{array}{c} m+s-1\\ l \end{array}\right]}_{p,q}p^{\frac{\left(m+s-l-1)(m+s-l-2\right)}{2}}q^{\frac{l(l-1)}{2}}{\left(\frac{q^{2}x}{p^{2}(1-x)}\right)}^{l}}{\prod_{j=0}^{m+s-2}\{p^{j-1}+q^{j-1}(\frac{q^{2}x}{p^{2}(1-x)})\}}\\ & = & \frac{2{\left[m+s\right]}_{p,q}qp^{4m+2s-3}}{{\left[2\right]}_{p,q}{\left[m\right]}_{p,q}^{2}}.\frac{\left(p^{m+s}(1-x)+q^{m+s}x\right)}{p(1-x+qx)}x. \end{array}$$

Thirdly, we deal with *B*3 as

15 $$\begin{array}{rcl} B3 & = & \frac{1}{{\left[3\right]}_{p,q}}\sum_{l=0}^{m+s}B_{m,l,s}^{p,q}(x)\frac{q^{2l}}{p^{2l-2m}{\left[m\right]}_{p,q}^{2}}\\ & = & \frac{p^{2m}}{{\left[3\right]}_{p,q}{\left[m\right]}_{p,q}^{2}}\sum_{l=0}^{m+s}\frac{{\left[\begin{array}{c} m+s\\ l \end{array}\right]}_{p,q}p^{\frac{\left(m+s-l)(m+s-l-1\right)}{2}}q^{\frac{l(l-1)}{2}}{\left(\frac{q^{2}x}{p^{2}(1-x)}\right)}^{l}{\left(1-x\right)}^{m+s}}{\prod_{j=0}^{m+s-2}\{p^{j+1}(1-x)+q^{j+1}x\}}\\ & = & \frac{p^{-2s}}{{\left[3\right]}_{p,q}{\left[m\right]}_{p,q}^{2}}.\frac{\left(p^{m+s}(1-x)+q^{m+s}x)(p^{m+s+1}(1-x)+q^{m+s+1}x\right)}{p(1-x)+qx}. \end{array}$$

As a consequence, $K_{m,s}^{(p,q)}(t^{2};x)$ is found as

$$\begin{aligned} K_{m,s}^{(p,q)} \bigl(t^{2};x \bigr) =& \frac{[m+s]_{p,q}p^{m-s+1}}{[m]^{2}_{p,q}}x+\frac {[m+s]_{p,q}[m+s-1]_{p,q}p^{2-2s}q^{2}}{[m]^{2}_{p,q}(p(1-x)+qx)}x^{2} \\ &{}+\frac{2[m+s]_{p,q}qp^{4m+2s-3}}{[m]^{2}_{p,q}[2]_{p,q}}.\frac {(p^{m+s}(1-x)+q^{m+s}x)}{p(1-x+qx)}x \\ &{}+\frac{p^{-2s}}{[3]_{p,q}[m]^{2}_{p,q}}.\frac {(p^{m+s}(1-x)+q^{m+s}x)(p^{m+s+1}(1-x)+q^{m+s+1}x)}{p(1-x)+qx}. \end{aligned}$$

If we reorganize, we obtain

16 $$\begin{aligned} K_{m,s}^{(p,q)} \bigl(t^{2};x \bigr) =& \frac {[m+s]_{p,q}[m+s-1]_{p,q}q^{2}p^{2-2s}}{[m]_{p,q}^{2}(p(1-x)+qx)}x^{2}+\frac {[m+s]_{p,q}p^{m-s+1}}{[m]_{p,q}^{2}}x \\ &{}+\frac {2[m+s]_{p,q}qp^{4m+2s-3}(p^{m+s}(1-x)+q^{m+s}x)}{[2]_{p,q}(p(1-x)+qx)[m]_{p,q}^{2}}x \\ &{}+\frac {p^{-2s}(p^{m+s}(1-x)+q^{m+s}x)(p^{m+s+1}(1-x)+q^{m+s+1}x)}{[3]_{p,q}[m]_{p,q}^{2}(p(1-x)+qx)} , \end{aligned}$$

as desired.

*(iv)* By using the linearity of the operators $K_{m,s}^{(p,q)}$, we acquire the first central moment $K_{m,s}^{(p,q)}(t-x;x)$ as

17 $$\begin{aligned} K_{m,s}^{(p,q)}(t-x;x) =&K_{m,s}^{(p,q)}(t;x)-xK_{m,s}^{(p,q)}(1;x) \\ =& \biggl( \frac{[m+s]_{p,q}}{[m]_{p,q}p^{s-1}} -\frac {p^{m}}{[2]_{p,q}[m]_{p,q}}+ \frac{q^{m+s}}{[2]_{p,q}[m]_{p,q}p^{s}}-1 \biggr)x \\ &{}+\frac{p^{m}}{[2]_{p,q}[m]_{p,q}}. \end{aligned}$$

*(v)* Similarly, we write the second central moment $K_{m,s}^{(p,q)}((t-x)^{2};x)$ as

18 $$ K_{m,s}^{(p,q)} \bigl((t-x)^{2};x \bigr)=K_{m,s}^{(p,q)} \bigl(t^{2};x \bigr)-2xK_{m,s}^{(p,q)}(t;x)+x^{2}K_{m,s}^{(p,q)}(1;x). $$

We now plug-in into equation (18) expressions (5), (6) and (7). Then we get

19 $$\begin{aligned} K_{m,s}^{(p,q)} \bigl((t-x)^{2};x \bigr) =& \biggl( \frac {[m+s]_{p,q}[m+s-1]_{p,q}q^{2}p^{2-2s}}{[m]_{p,q}^{2}(p(1-x)+qx)} \\ &{}+\frac {-2[2]_{p,q}[m+s]_{p,q}p^{1-s}+2p^{m}-2q^{m+s}p^{-s}}{[2]_{p,q}[m]_{p,q}}+1 \biggr) x^{2} \\ &{}+ \biggl(\frac{[m+s]_{p,q}p^{m-s+1}}{[m]_{p,q}^{2}} +\frac {2[m+s]_{p,q}qp^{4m+2s-3}(p^{m+s}(1-x)+q^{m+s}x)}{[2]_{p,q}[m]_{p,q}^{2}(p(1-x)+qx)} \\ &{}-\frac {2p^{m}}{[2]_{p,q}[m]_{p,q}} \biggr)x \\ &{}+ \frac {p^{-2s}(p^{m+s}(1-x)+q^{m+s}x)(p^{m+s+1}(1-x)+q^{m+s+1}x)}{[3]_{p,q}(p(1-x)+qx)[m]_{p,q}^{2}}. \end{aligned}$$

 □

We can easily see that $K_{m,s}^{(p,q)}(f;x)$ are linear positive operators.

### Remark 1

[15] Let *p*, *q* satisfy $0< q< p \leq1$ and $\lim_{m\rightarrow\infty}[m]_{p,q}=\frac{1}{p-q}$. To obtain the convergence results for operators $K_{m,s}^{(p,q)}(f;x)$, we take sequences $q_{m}\in(0,1)$, $p_{m}\in (q_{m},1]$ such that $\lim_{m\rightarrow\infty}p_{m}=1$, $\lim_{m\rightarrow\infty}q_{m}=1$, $\lim_{m\rightarrow\infty}p_{m}^{m}=1$ and $\lim_{m\rightarrow\infty}q_{m}^{m}=1$. Such sequences can be constructed by taking $p_{m}=1-1/m^{2}$ and $q_{m}=1-1/2m^{2}$.

Now we will present the next theorem, which ensures the approximation process according to Korovkin’s approximation theorem.

### Theorem 1

*Let* $K_{m,s}^{(p,q)}(f;x)$ *satisfy the conditions* $p_{m}\rightarrow1$, $q_{m}\rightarrow1$, $p_{m}^{m}\rightarrow1$ *and* $q_{m}^{m}\rightarrow1$ *as* $m\rightarrow\infty$ *for* $q_{m}\in(0,1)$, $p_{m}\in(q_{m},1]$. *Then for every monotone increasing function* $f\in C [ 0,s+1 ]$, *operators* $K_{m,s}^{(p,q)}(f;x)$ *converge uniformly to* *f*.

### Proof

By the Korovkin theorem, it is sufficient to prove that

$$ \lim_{m\longrightarrow\infty} \bigl\Vert K_{m,s}^{(p,q)}e_{k}-e_{k} \bigr\Vert =0,\quad k=0,1,2, $$

where $e_{k}(x)=x^{k}$, $k=0,1,2$.

*(i)* By using Eq. (5), it can be clearly seen that

$$ \lim_{m\longrightarrow\infty} \bigl\Vert K_{m,s}^{(p,q)}e_{0}-e_{0} \bigr\Vert =\lim_{m\longrightarrow\infty}\sup_{x \in[0,1] } \bigl\vert K_{m,s}^{(p,q)}(1;x)-1 \bigr\vert =0. $$

*(ii)* By Eq. (6), we write

$$\begin{aligned}& \lim_{m\longrightarrow\infty} \bigl\Vert K_{m,s}^{(p,q)}e_{1}-e_{1} \bigr\Vert \\& \quad = \lim_{m\longrightarrow\infty}\sup_{x \in[0,1] } \bigl\vert K_{m,s}^{(p,q)}(t;x)-x \bigr\vert \\& \quad = \lim_{m\longrightarrow\infty}\sup_{x \in[0,1] } \biggl\vert \biggl( \frac{[m+s]_{p,q}}{p^{s-1}[m]_{p,q}} -\frac {p^{m}}{[2]_{p,q}[m]_{p,q}}+ \frac{q^{m+s}}{[2]_{p,q}[m]_{p,q}p^{s}}-1 \biggr)x +\frac{p^{m}}{[2]_{p,q}[m]_{p,q}} \biggr\vert \\& \quad \leq \lim_{m\longrightarrow\infty} \biggl( \frac {[m+s]_{p,q}}{p^{s-1}[m]_{p,q}}-1+ \frac{q^{m+s}}{[2]_{p,q}[m]_{p,q}p^{s}} \biggr) \\& \quad = 0. \end{aligned}$$

*(iii)* From Eq. (7), we have

$$\begin{aligned}& \lim_{m\longrightarrow\infty} \bigl\Vert K_{m,s}^{(p,q)}e_{2}-e_{2} \bigr\Vert \\& \quad = \lim_{m\longrightarrow\infty}\sup_{x \in[0,1] } \bigl\vert K_{m,s}^{(p,q)} \bigl(t^{2};x \bigr)-x^{2} \bigr\vert \\& \quad = \lim_{m\longrightarrow\infty}\sup_{x \in[0,1] } \biggl\vert \biggl( \frac {[m+s]_{p,q}[m+s-1]_{p,q}q^{2}p^{2-2s}}{[m]_{p,q}^{2}(p(1-x)+qx)}-1 \biggr) x^{2} \\& \qquad {}+\frac{[m+s]_{p,q}p^{m-s+1}}{[m]_{p,q}^{2}}x +\frac {2[m+s]_{p,q}qp^{4m+2s-3}(p^{m+s}(1-x)+q^{m+s}x)}{[2]_{p,q}[m]_{p,q}^{2}(p(1-x)+qx)}x \\& \qquad {}+\frac {p^{-2s}(p^{m+s}(1-x)+q^{m+s}x)(p^{m+s+1}(1-x)+q^{m+s+1}x)}{[3]_{p,q}[m]_{p,q}^{2}(p(1-x)+qx)} \biggr\vert \\& \quad \leq \lim_{m\longrightarrow\infty} \biggl( \biggl( \frac {[m+s]_{p,q}[m+s-1]_{p,q}q^{2}p^{2-2s}}{[m]_{p,q}^{2}(p(1-x)+qx)}-1 \biggr) +\frac{[m+s]_{p,q}p^{m-s+1}}{[m]_{p,q}^{2}} \\& \qquad {}+\frac {2[m+s]_{p,q}qp^{4m+2s-3}(p^{m+s}(1-x)+q^{m+s}x)}{[2]_{p,q}[m]_{p,q}^{2}(p(1-x)+qx)} \\& \qquad {}+\frac {p^{-2s}(p^{m+s}(1-x)+q^{m+s}x)(p^{m+s+1}(1-x)+q^{m+s+1}x)}{[3]_{p,q}[m]_{p,q}^{2}(p(1-x)+qx)} \biggr) \\& \quad = 0. \end{aligned}$$

Consequently, the proof is finished. □

Before mentioning local approximation properties, we will give two lemmas as follows.

### Lemma 2

*If* *f* *is a monotone increasing function*, *then the constructed operators* $K_{m,s}^{(p,q)}(f;x)$ *are linear and positive*.

### Lemma 3

*Let* $0< q< p\leq1$, $0< u< v$, *and* $\frac{1}{u }+\frac{1}{v }=1$. *Then the operators* $K_{m,s}^{(p,q)} ( f;x )$ *satisfy the following Hölder inequality*:

$$ K_{m,s}^{(p,q)} \bigl( \vert fg \vert ;x \bigr) \leq \bigl( K_{m,s}^{(p,q)} \bigl( \vert f \vert ^{u };x \bigr) \bigr) ^{\frac{1}{u }} \bigl( K_{m,s}^{(p,q)} \bigl( \vert g \vert ^{v };x \bigr) \bigr) ^{\frac{1}{v }}. $$

## Local approximation properties

Let *f* be a continuous function on $C[0,s+1]$. The modulus of continuity of *f* is denoted by $w(f,\sigma ) $ and given as

20 $$ w(f,\sigma)=\sup_{\overset{ \vert y-x \vert \leq \sigma}{x,y\in [ 0,1 ] }} \bigl\vert f ( y ) -f ( x ) \bigr\vert . $$

Then we know from the properties of modulus of continuity that for each $\sigma>0$, we have

21 $$ \bigl\vert f ( y ) -f ( x ) \bigr\vert \leq w(f,\sigma) \biggl( \frac{ \vert y-x \vert }{\sigma}+1 \biggr), \quad x,y\in[0,1]. $$

And also, for $f\in C[0,s+1]$ we have $\lim_{\sigma\rightarrow 0^{+}}w(f,\sigma)=0$. First of all, we begin by giving the rate of convergence of the operators $K_{m,s}^{(p,q)}(f;x)$ by using the modulus of continuity.

### Theorem 2

*Let the sequences* $p:= ( p_{m} ) $ *and* $q:= ( q_{m} ) $, $0< q_{m}< p_{m}\leq1$, *satisfy the conditions* $p_{m}\rightarrow1$, $q_{m}\rightarrow1$, $p_{m}^{m}\rightarrow1$ *and* $q_{m}^{m}\rightarrow1$ *as* $m\rightarrow\infty$. *Then for each* $f\in C [ 0,s+1 ]$,

$$ \bigl\lVert K_{m,s}^{(p,q)}f-f \bigr\lVert _{C[0,s+1]}\leq2\omega \bigl( f;\sigma_{m} ( x ) \bigr), $$

*where*

22 $$ \sigma_{m} ( x )=\sqrt{ K_{m,s}^{(p,q)} \bigl((t-x)^{2};x \bigr)} $$

*and* $K_{m,s}^{(p,q)}((t-x)^{2};x)$ *is as given by* (19).

### Proof

By the positivity and linearity of the operators $K_{m,s}^{(p,q)}(f;x)$, we get

$$\begin{aligned} \bigl\vert K_{m,s}^{(p,q)}(f;x) -f ( x ) \bigr\vert =& \bigl\vert K_{m,s}^{(p,q)} \bigl(f(t)-f(x);x \bigr) \bigr\vert \\ \leq& K_{m,s}^{(p,q)} \bigl( \bigl\vert f ( t ) -f ( x ) \bigr\vert ;q;x \bigr). \end{aligned}$$

After that we apply (21) and obtain

23 $$\begin{aligned} \bigl\vert K_{m,s}^{(p,q)}(f;x) -f ( x ) \bigr\vert \leq& K_{m,s}^{(p,q)} \biggl( w(f,\sigma_{m}) \biggl( \frac{ \vert t-x \vert }{\sigma_{m} }+1 \biggr);x \biggr) \\ =& \frac{ w(f,\sigma_{m})}{\sigma_{m}}\sqrt{ K_{m,s}^{(p,q)} \bigl((t-x)^{2};x \bigr)}+w(f,\sigma_{m}) \\ =&w(f,\sigma_{m}) \biggl(1+\frac{1}{\sigma_{m}}\sqrt{ K_{m,s}^{(p,q)} \bigl((t-x)^{2};x \bigr)} \biggr). \end{aligned}$$

Then, taking supremum of the last equation, we have

$$\begin{aligned} \bigl\lVert K_{m,s}^{(p,q)}f-f \bigr\lVert =&\sup _{x \in[0,1] } \bigl\vert K_{m,s}^{(p,q)}(f;x) -f ( x ) \bigr\vert \\ \leq&w(f,\sigma_{m}) \biggl(1+\frac{1}{\sigma_{m}}\sqrt{ K_{m,s}^{(p,q)} \bigl((t-x)^{2};x \bigr)} \biggr). \end{aligned}$$

Choose

$$\begin{aligned} \sigma_{m} ( x ) =& \biggl\lbrace \biggl( \frac {q^{2}[m+l]_{p,q}[m+l-1]_{p,q}}{([m]_{p,q}+\beta)^{2}(p(1-x)+qx)}- \frac{2[m+l]_{p,q}}{[m]_{p,q}+\beta}+1 \biggr) x^{2} \\ &{}+ \biggl( -\frac{2\alpha}{[m]_{p,q}+\beta}+ \frac{[m+l]_{p,q} ( p^{m+l-1}+2\alpha)}{([m]_{p,q}+\beta)^{2}} \biggr) x + \biggl( \frac{\alpha}{[m]_{p,q}+\beta} \biggr)^{2} \biggr\rbrace ^{1/2}. \end{aligned}$$

Thus, we achieve

$$ \bigl\lVert K_{m,s}^{(p,q)}f-f \bigr\lVert _{C[0,s+1]}\leq2\omega \bigl( f;\sigma_{m} ( x ) \bigr). $$

This result completes the proof of the theorem. □

In what follows, by using Lipschitz functions, we will give the rate of convergence of the operators $K_{m,s}^{(p,q)}(f;x)$. We remember that if the inequality

24 $$ \bigl\vert f ( y ) -f ( x ) \bigr\vert \leq M \vert y-x \vert ^{\alpha} ;\quad \forall x,y\in{}[ 0,1] $$

is satisfied, then *f* belongs to the class $\mathrm{Lip}_{M} ( \alpha )$.

### Theorem 3

*Denote* $p:= ( p_{m} ) $ *and* $q:= ( q_{m} ) $ *satisfying* $0< q_{m}< p_{m}\leq1$. *Then*, *for every* $f\in \mathrm{Lip}_{M} ( \alpha ) $, *we have*

$$ \bigl\lVert K_{m,s}^{(p,q)}f-f \bigr\rVert \leq M\sigma _{m}^{\alpha} ( x ), $$

*where* $\sigma_{m}(x)$ *is the same as in* (22).

### Proof

Let *f* belong to the class $\mathrm{Lip}_{M} ( \alpha ) $ for some $0<\alpha \leq1$. Using the monotonicity of the operators $K_{m,s}^{(p,q)}(f;x) $ and (24), we obtain

$$\begin{aligned} \bigl\vert K_{m,s}^{(p,q)}(f;x) -f ( x ) \bigr\vert \leq& K_{m,s}^{(p,q)} \bigl( \bigl\vert f ( t ) -f ( x ) \bigr\vert ;x \bigr) \\ \leq&M K_{m,s}^{(p,q)} \bigl( \vert t-x \vert ^{\alpha};x \bigr). \end{aligned}$$

Taking $p=\frac{2}{\alpha}$, $q=\frac{2}{2-\alpha}$ and applying Hölder inequality yields

$$\begin{aligned} \bigl\vert K_{m,s}^{(p,q)} ( f;x ) -f ( x ) \bigr\vert \leq &M \bigl\{ K_{m,s}^{(p,q)} \bigl( (t-x ) ^{2};x \bigr) \bigr\} ^{\frac{\alpha}{2}} \\ \leq&M\sigma_{m}^{\alpha} ( x ). \end{aligned}$$

By choosing $\sigma_{m} ( x )$ as in Theorem 2, we complete the proof as desired. □

Finally, in the light of Peetre-K functionals, we obtain the rate of convergence of the constructed operators $K_{m,s}^{(p,q)}(f;x)$. We recall the properties of Peetre-K functionals, which are defined as

$$ K(f,\delta):=\inf_{g \in C^{2}[0,s+1] } \bigl\{ \Vert f-g \Vert _{C [ 0,s+1 ]}+\delta \Vert g \Vert _{C^{2} [ 0,s+1 ]} \bigr\} . $$

Here $C^{2} [ 0,s+1 ]$ defines the space of the functions *f* such that $f, f', f''\in C[0,s+1]$. The norm in this space is given by

$$ \Vert f \Vert _{C^{2} [ 0,s+1 ] }=\bigl\lVert f''\bigr\lVert _{C [ 0,s+1 ] } +\bigl\lVert f' \bigr\lVert _{C [ 0,s+1 ] }+\lVert f \lVert _{C [ 0,s+1 ] }. $$

Also we consider the second modulus of smoothness of $f\in C[0,s+1]$, namely

$$ \omega_{2}(f,\delta):=\sup_{0< h< \delta} \sup _{x,x+h\in[0,s+1]} \bigl\vert f(x+2h)-2f(x+h)+f(x) \bigr\vert ,\quad \delta>0. $$

We know from [7] that for $M>0$

$$ K(f,\delta)\leq M\omega_{2}(f,\sqrt{\sigma}). $$

Before giving the main theorem, we present an auxiliary lemma, which will be used in the proof of the theorem.

### Lemma 4

*For any* $f\in C[0,s+1]$, *we have*

25 $$\begin{aligned} \bigl\vert K_{m,s}^{(p,q)}(f;x) \bigr\vert \leq \Vert f \Vert . \end{aligned}$$

### Proof

$$\begin{aligned} \bigl\vert K_{m,s}^{(p,q)}(f;x) \bigr\vert =& \Biggl\vert \sum_{l=0}^{m+s}B_{m,l,s}^{p,q}(x) \int_{0}^{1}f \biggl( \frac{p[l]_{p,q}+q^{l}t}{p^{l-m}[m]_{p,q}} \biggr) \,d_{p,q}t \Biggr\vert \\ \leq& \sum_{l=0}^{m+s} B_{m,l,s}^{p,q}(x) \biggl\vert \int_{0}^{1}f \biggl( \frac{p[l]_{p,q}+q^{l}t}{p^{l-m}[m]_{p,q}} \biggr) \,d_{p,q}t \biggr\vert \\ \leq& \sum_{l=0}^{m+s} B_{m,l,s}^{p,q}(x) \int_{0}^{1} \biggl\vert f \biggl( \frac{p[l]_{p,q}+q^{l}t}{p^{l-m}[m]_{p,q}} \biggr) \biggr\vert \,d_{p,q}t \\ \leq& \Vert f \Vert K_{m,s}^{(p,q)} (1;x) \\ =& \Vert f \Vert . \end{aligned}$$

 □

### Theorem 4

*Let* $0< q_{m}< p_{m}\leq1$, $m\in\mathbb{N}$ *and* $f\in C[0,s+1] $. *There exists a constant* $M>0$ *such that*

$$ \bigl\vert K_{m,s}^{(p,q)}(f;x)-f ( x ) \bigr\vert \leq M \omega_{2} \bigl(f,\alpha_{m}(x) \bigr)+\omega \bigl(f, \beta_{m}(x) \bigr), $$

*where*

26 $$\begin{aligned} \alpha_{m} ( x )= \sqrt{ K_{m,s}^{(p,q)} \bigl((t-x)^{2};x \bigr)+\frac{1}{2} \biggl( \frac {([2]_{p,q}[m+s]_{p,q}p^{1-s}-p^{m}+p^{-s}q^{m+s})x+p^{m} }{[2]_{p,q}[m]_{p,q}}-x \biggr)^{2} } \end{aligned}$$

*and*

27 $$\begin{aligned} \beta_{m}(x)= \frac{([2]_{p,q}[m+s]_{p,q}p^{1-s}-p^{m}+p^{-s}q^{m+s})x+p^{m} }{[2]_{p,q}[m]_{p,q}}-x. \end{aligned}$$

### Proof

Define an auxiliary operator $K_{m,s}^{*}$ as follows:

28 $$\begin{aligned} K_{m,s}^{*}(f;x)=K_{m,s}^{(p,q)}(f;x)-f \biggl( \frac{([2]_{p,q}[m+s]_{p,q}p^{1-s}-p^{m}+p^{-s}q^{m+s})x+p^{m} }{[2]_{p,q}[m]_{p,q}} \biggr)+f(x). \end{aligned}$$

From Lemma 1, we have

29 $$\begin{aligned}& K_{m,s}^{*}(1;x)=1, \\& \begin{aligned}[b] K_{m,s}^{*}(t-x;x)&= K_{m,s}^{(p,q)} \bigl((t-x);x \bigr)- \biggl( \frac {([2]_{p,q}[m+s]_{p,q}p^{1-s}-p^{m}+p^{-s}q^{m+s})x+p^{m} }{[2]_{p,q}[m]_{p,q}}-x \biggr)\hspace{-20pt} \\ &= \biggl( \frac{[m+s]_{p,q}}{p^{s-1}[m]_{p,q}} -\frac {p^{m}}{[2]_{p,q}[m]_{p,q}}+ \frac{q^{m+s}}{[2]_{p,q}[m]_{p,q}p^{s}}-1 \biggr)x+x \\ &\quad {}+\frac{p^{m}}{[2]_{p,q}[m]_{p,q}}-\frac {([2]_{p,q}[m+s]_{p,q}p^{1-s}-p^{m}+p^{-s}q^{m+s})x+p^{m} }{[2]_{p,q}[m]_{p,q}}\\ &=0. \end{aligned} \end{aligned}$$

Taylor’s expansion for a function $g \in C^{2}[0,s+1]$ can be written as follows:

30 $$\begin{aligned} g(t)=g(x)+(t-x)g'(x)+ \int_{x}^{t}(t-u)g''(u)\,du, \quad t\in[0,1]. \end{aligned}$$

Then applying operator $K_{m,s}^{*} $ to both sides of (30), we get

$$\begin{aligned} K_{m,s}^{*}(g;x) =&K_{m,s}^{*} \biggl(g(x)+(t-x)g'(x)+ \int_{x}^{t}(t-u)g''(u)\,du \biggr) \\ =&g(x)+K_{m,s}^{*} \bigl((t-x)g'(x);x \bigr)+K_{m,s}^{*} \biggl( \int_{x}^{t}(t-u)g''(u)\,du \biggr). \end{aligned}$$

So,

$$ K_{m,s}^{*}(g;x)-g(x)=g'(x)K_{m,s}^{*} \bigl((t-x);x \bigr)+K_{m,s}^{*} \biggl( \int_{x}^{t}(t-u)g''(u)\,du \biggr). $$

Using (29) and (28), we obtain

31 $$\begin{aligned}& K_{m,s}^{*}(g;x)-g(x) \\& \quad =K_{m,s}^{*} \biggl( \int_{x}^{t}(t-u)g''(u)\,du \biggr) \\& \quad = K_{m,s}^{(p,q)} \biggl( \int_{x}^{t}(t-u)g''(u)\,du \biggr) \\& \qquad {}- \int_{x}^{\frac{([2]_{p,q}[m+s]_{p,q}p^{1-s}-p^{m}+p^{-s}q^{m+s})x+p^{m} }{[2]_{p,q}[m]_{p,q}}} \biggl(\frac {([2]_{p,q}[m+s]_{p,q}p^{1-s}-p^{m}+p^{-s}q^{m+s})x+p^{m} }{[2]_{p,q}[m]_{p,q}} \\& \qquad {}-u \biggr)g''(u)\,du \\& \qquad {}+ \int_{x}^{x} \biggl(\frac {([2]_{p,q}[m+s]_{p,q}p^{1-s}-p^{m}+p^{-s}q^{m+s})x+p^{m} }{[2]_{p,q}[m]_{p,q}}-u \biggr)g''(u)\,du. \end{aligned}$$

Moreover,

32 $$ \biggl\vert \int_{x}^{t}(t-u)g''(u)\,du \biggr\vert \leq \int_{x}^{t} \vert t-u \vert \bigl\vert g''(u) \bigr\vert \,du \leq \bigl\Vert g'' \bigr\Vert \int_{x}^{t} \vert t-u \vert \,du \leq (t-x)^{2} \bigl\Vert g'' \bigr\Vert $$

and

33 $$\begin{aligned}& \biggl\vert \int_{x}^{\frac{([2]_{p,q}[m+s]_{p,q}p^{1-s}-p^{m}+p^{-s}q^{m+s})x+p^{m} }{[2]_{p,q}[m]_{p,q}}} \biggl(\frac {([2]_{p,q}[m+s]_{p,q}p^{1-s}-p^{m}+p^{-s}q^{m+s})x+p^{m} }{[2]_{p,q}[m]_{p,q}} \\& \qquad {}-u \biggr)g''(u)\,du \biggr\vert \\& \quad \leq \bigl\Vert g'' \bigr\Vert \int_{x}^{\frac{([2]_{p,q}[m+s]_{p,q}p^{1-s}-p^{m}+p^{-s}q^{m+s})x+p^{m} }{[2]_{p,q}[m]_{p,q}}} \biggl(\frac {([2]_{p,q}[m+s]_{p,q}p^{1-s}-p^{m}+p^{-s}q^{m+s})x+p^{m} }{[2]_{p,q}[m]_{p,q}} \\& \qquad {}-u \biggr)\,du \\& \quad = \frac{ \Vert g'' \Vert }{2} \biggl( \frac {([2]_{p,q}[m+s]_{p,q}p^{1-s}-p^{m}+p^{-s}q^{m+s})x+p^{m} }{[2]_{p,q}[m]_{p,q}}-x \biggr)^{2}. \end{aligned}$$

Let us employ (32) and (33) when taking the absolute value of (31). We obtain

$$\begin{aligned} \bigl\vert K_{m,s}^{*}(g;x)-g(x) \bigr\vert \leq& \bigl\Vert g'' \bigr\Vert K_{m,s}^{(p,q)} \bigl((t-x)^{2};x \bigr) \\ &{}+\frac{ \Vert g'' \Vert }{2} \biggl( \frac {([2]_{p,q}[m+s]_{p,q}p^{1-s}-p^{m}+p^{-s}q^{m+s})x+p^{m} }{[2]_{p,q}[m]_{p,q}}-x \biggr)^{2} \\ =& \bigl\Vert g'' \bigr\Vert \alpha_{m}^{2}(x), \end{aligned}$$

where

34 $$\begin{aligned}& \alpha_{m} ( x ) \\& \quad =\sqrt{ K_{m,s}^{(p,q)} \bigl((t-x)^{2};x \bigr)+\frac{1}{2} \biggl( \frac {([2]_{p,q}[m+s]_{p,q}p^{1-s}-p^{m}+p^{-s}q^{m+s})x+p^{m} }{[2]_{p,q}[m]_{p,q}}-x \biggr)^{2} }. \end{aligned}$$

We now give an upper bound for the auxiliary operator $K_{m,l,p,q}^{*}(f;x)$. From Lemma 4 we get

$$\begin{aligned} \bigl\vert K_{m,s}^{*}(f;x) \bigr\vert =& \biggl\vert K_{m,s}^{(p,q)}(f;x)-f \biggl( \frac {([2]_{p,q}[m+s]_{p,q}p^{1-s}-p^{m}+p^{-s}q^{m+s})x+p^{m} }{[2]_{p,q}[m]_{p,q}} \biggr)+f(x) \biggr\vert \\ \leq& \bigl\vert K_{m,s}^{(p,q)}(f;x) \bigr\vert + \biggl\vert f \biggl( \frac {([2]_{p,q}[m+s]_{p,q}p^{1-s}-p^{m}+p^{-s}q^{m+s})x+p^{m} }{[2]_{p,q}[m]_{p,q}} \biggr) \biggr\vert + \bigl\vert f(x) \bigr\vert \\ \leq&3 \Vert f \Vert . \end{aligned}$$

Accordingly,

35 $$\begin{aligned}& \begin{aligned} &\bigl\vert K_{m,s}^{(p,q)}(f;x)-f(x) \bigr\vert \\ &\quad = \biggl\vert K_{m,s}^{*}(f;x)-f(x)+f \biggl( \frac {([2]_{p,q}[m+s]_{p,q}p^{1-s}-p^{m}+p^{-s}q^{m+s})x+p^{m} }{[2]_{p,q}[m]_{p,q}} \biggr)-f(x) \\ &\qquad {} \mp g(x)\mp K_{m,s}^{*}(g;x) \biggr\vert , \end{aligned} \\& \begin{aligned}[b] &\bigl\vert K_{m,s}^{(p,q)}(f;x)-f(x) \bigr\vert \\ &\quad \leq \bigl\vert K_{m,s}^{*}(f-g;x)-(f-g) (x) \bigr\vert + \bigl\vert K_{m,s}^{*}(g;x)-g(x) \bigr\vert \\ &\qquad {}+ \biggl\vert f \biggl( \frac {([2]_{p,q}[m+s]_{p,q}p^{1-s}-p^{m}+p^{-s}q^{m+s})x+p^{m} }{[2]_{p,q}[m]_{p,q}} \biggr) -f(x) \biggr\vert \\ &\quad \leq4 \Vert f-g \Vert + \bigl\Vert g'' \bigr\Vert \alpha_{m}^{2}(x)+\omega \bigl(f,\beta_{m}(x) \bigr) \biggl(\frac{ ( \frac {([2]_{p,q}[m+s]_{p,q}p^{1-s}-p^{m}+p^{-s}q^{m+s})x+p^{m} }{[2]_{p,q}[m]_{p,q}}-x )}{\beta_{m}(x)}+1 \biggr)\hspace{-20pt} \\ &\quad =4 \Vert f-g \Vert + \bigl\Vert g'' \bigr\Vert \alpha_{m}^{2}(x) \\ &\qquad {}+2\omega \biggl(f, \biggl( \frac {([2]_{p,q}[m+s]_{p,q}p^{1-s}-p^{m}+p^{-s}q^{m+s})x+p^{m} }{[2]_{p,q}[m]_{p,q}}-x \biggr) \biggr), \end{aligned} \end{aligned}$$

where

36 $$ \beta_{m}(x)= \frac{([2]_{p,q}[m+s]_{p,q}p^{1-s}-p^{m}+p^{-s}q^{m+s})x+p^{m} }{[2]_{p,q}[m]_{p,q}}-x. $$

Finally, for all $g\in C^{2}[0,s+1]$, taking the infimum of (35), we get

37 $$ \bigl\vert K_{m,s}^{(p,q)}(f;x)-f(x) \bigr\vert \leq4K \bigl(f,\alpha_{m}^{2}(x) \bigr)+\omega \bigl(f, \beta_{m}(x) \bigr). $$

Consequently, using the property of Peetre-K functional, we obtain

38 $$ \bigl\vert K_{m,s}^{(p,q)}(f;x)-f(x) \bigr\vert \leq M \omega_{2} \bigl(f,\alpha_{m}(x) \bigr)+\omega \bigl(f, \beta_{m}(x) \bigr). $$

This completes the proof. □

## Graphical illustrations

In this section, we illustrate an approximation of the operators $K_{m,s}^{(p,q)}$ for a function $f(x)$ by employing Matlab codes. Let us specially choose

$$f(x)= \frac{1}{96}\tan\biggl(\frac{x}{16}\biggr) \biggl( \frac{x}{8}\biggr)^{2}\biggl(1-\frac{x}{4} \biggr)^{3}, $$

and take $p=0.8$, $q=0.7$ and $s=5$.

### Algorithm 1



### Algorithm 2



Initially, we discuss the error estimates of the Kantorovich type Lupaş–Schurer operators based on $(p, q)$-integers for different values of *x* and *m* in Table 1 by using Algorithm 1.

**Table 1:** Error estimates for different values of *x* when $s=5$, $p=0.8$ and $q=0.7$

*m*	Error at *x* = 0.1	Error at *x* = 0.5	Error at *x* = 0.9
5	0.1494⋅10^−6^	0.0583⋅10^−6^	0.0441⋅10^−6^
10	0.0326⋅10^−6^	0.3298⋅10^−6^	0.1599⋅10^−6^
15	0.0135⋅10^−6^	0.2398⋅10^−6^	0.0078⋅10^−6^

**Figure 1:**
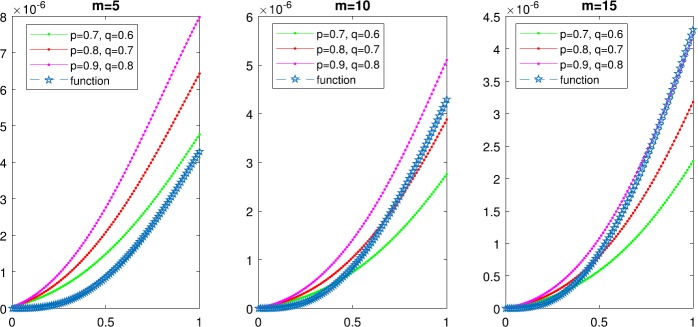
Convergence of $(p, q)$-analogue Lupaş–Schurer–Kantorovich operators $K_{m,s}^{(p,q)}(f;x)$ for various values of $p,1$ and *m* with fixed $s=5$

**Figure 2:**
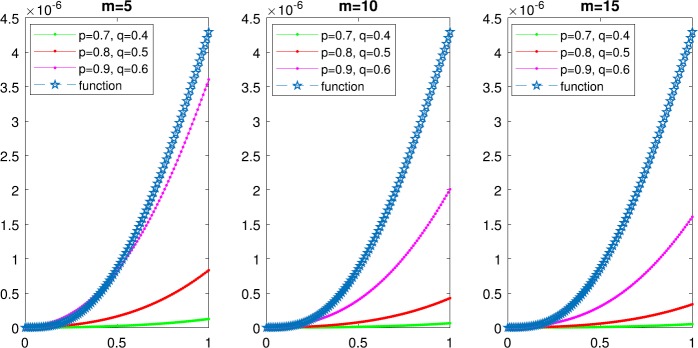
Convergence of the $(p, q)$-analogue Lupaş–Schurer operators $L_{m,l}^{p,q}(f;x)$ with fixed $l=5$ for various values of *p* and *q*

And then, we illustrate the convergence of the $(p, q)$-Lupaş–Schurer–Kantorovich operators $K_{m,s}^{(p,q)}(f;x)$ for the selected function $f(x)= \frac{1}{96}\tan(\frac{x}{16})(\frac {x}{8})^{2}(1-\frac{x}{4})^{3}$ in Fig. 1 for several values of *m* by using Algorithm 2. Furthermore, we give the error estimates in Table 2 in order to indicate that the $(p, q)$-analogue Lupaş–Schurer operators [14] converge and then plot Fig. 2. It can be clearly seen that the $(p, q)$-Lupaş–Schurer–Kantorovich operators converge faster than the $(p, q)$-analogue Lupaş–Schurer operators.

**Table 2:** Error estimates of $(p, q)$-Lupaş–Schurer operators for various values of *x*

*m*	Error at *x* = 0.1	Error at *x* = 0.5	Error at *x* = 0.9
5	0.0067⋅10^−5^	0.3011⋅10^−5^	0.4464⋅10^−5^
10	0.0073⋅10^−5^	0.3821⋅10^−5^	0.5743⋅10^−5^
15	0.0075⋅10^−5^	0.4077⋅10^−5^	0.6141⋅10^−5^

## Conclusion

In this paper, we constructed a new kind of Lupaş operators based on $(p, q)$-integers to provide a better error estimation. Firstly, we investigated some local approximation results by the help of the well-known Korovkin theorem. Also, we calculated the rate of convergence of the constructed operators employing the modulus of continuity, by using Lipschitz functions and then with the help of Peetre’s K-functional. Additionally, we presented a table of error estimates of the $(p, q)$-Lupaş–Schurer–Kantorovich operators for a certain function. Finally, we compared the convergence of the new operator to that of the $(p, q)$-analogue of Lupaş–Schurer operator.
